# P-1671. Variation in clinical practice and attitudes on antimicrobial prophylaxis for patients undergoing chimeric antigen receptor (CAR) T-cell therapy for hematologic malignancies: a survey of cancer centers across the United States

**DOI:** 10.1093/ofid/ofae631.1837

**Published:** 2025-01-29

**Authors:** Susan K Seo, Nina Cohen, Sanjeet S Dadwal, Zeinab El Boghdadly, Hannah Imlay, Elizabeth M Krantz, Jerod Nagel, Erica J Stohs, Catherine Liu

**Affiliations:** Memorial Sloan Kettering, New York, New York; Memorial Sloan Kettering Cancer Center, New York, NY; City of Hope National Medical Center, Duarte, CA; The Ohio State University, Columbus, OH; University of Utah Health, Salt Lake City, UT; Fred Hutch Cancer Center, Seattle, Washington; Michigan Medicine, Ann Arbor, Michigan; University of Nebraska Medical Center, Omaha, Nebraska; Fred Hutchinson Cancer Center, Seattle, WA

## Abstract

**Background:**

Adults with hematologic malignancies undergoing chimeric antigen receptor (CAR) T-cell therapy are at risk for infections, but there is no consensus approach to antimicrobial prophylaxis (ppx). The objective was to assess clinical practice and attitudes on antimicrobial ppx in adult CAR T-cell patients (pts) across United States (US) cancer centers.

**Figure:**
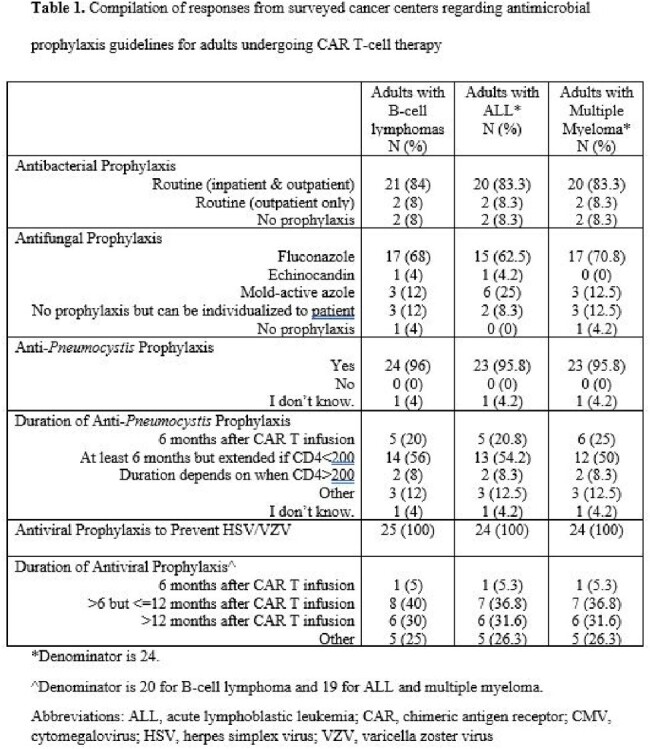

**Methods:**

A 17-item voluntary electronic survey was developed and distributed via email to Infectious Disease physicians and pharmacists at US cancer centers between 9/11/23 and 10/1/23.

**Figure:**
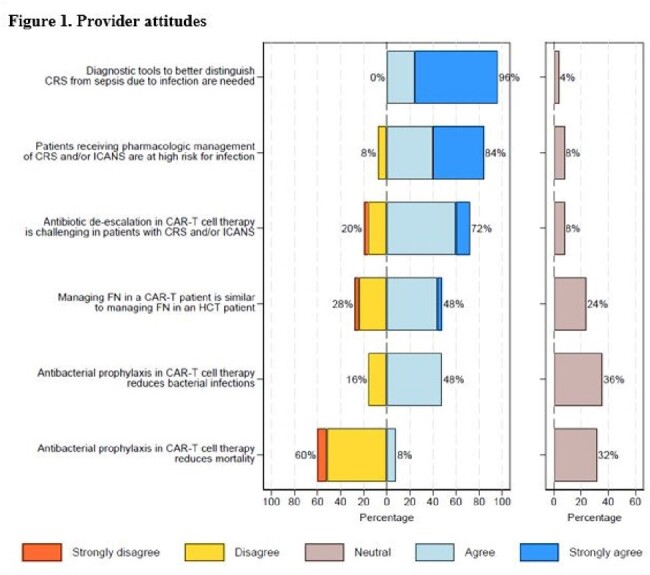

**Results:**

Responses from 25 (32.9%) of 76 cancer centers were analyzed. All responding centers had guidelines for pts with B-cell lymphomas (BCL), acute lymphoblastic leukemia (ALL), and multiple myeloma (MM) (Table 1). Routine antibacterial ppx was specified in 92% (23/25) BCL, 91.7% (22/24) ALL, and 91.7% (22/24) MM guidelines. Antifungal ppx was recommended in 84% (21/25) BCL, 91.7% (22/24) ALL, and 83.3% (20/24) MM guidelines. Fluconazole was most commonly given for BCL (17/25, 68%), ALL (15/24, 62.5%), and MM (17/24, 70.8%) pts. Anti-*Pneumocystis* ppx was provided in 96% (24/25) BCL, 95.8% (23/24) ALL, and 95.8% (23/24) MM guidelines. Acyclovir was routinely given to all BCL, ALL, and MM pts. Variability in duration of anti-*Pneumocysti*s and antiviral ppx was seen (Table 1). Fifteen of 25 (60%) respondents reported cytomegalovirus monitoring after CAR T-cell infusion for fever workup (N=7) or in select high-risk pts (N=8). Seventeen of 25 (68%) had guidelines for intravenous immunoglobulin repletion. Majority (24/25, 96%) agreed that diagnostic tools distinguishing cytokine release syndrome (CRS) from sepsis were needed, and 84% (21/25) agreed that pts receiving pharmacologic management for CRS and/or immune effector cell-associated neurotoxicity syndrome were at high risk for infection (Figure 1). Only 48% (12/25) felt that antibacterial ppx reduced bacterial infections, and only 8% (2/25) felt that antibacterial ppx reduced mortality.

**Conclusion:**

Variations in antimicrobial ppx guidelines for adults undergoing CAR T-cell therapy for hematologic malignanices among responding US cancer centers were observed. Few respondents perceived improved patient outcomes with antibacterial ppx.

**Disclosures:**

**Susan K. Seo, MD**, Merck: Grant/Research Support **Sanjeet S. Dadwal, MD, FACP, FIDSA**, Allovir: Advisor/Consultant|Allovir: Grant/Research Support|Ansun Biopharma: Advisor/Consultant|Ansun Biopharma: Grant/Research Support|Aseptiscope: Advisor/Consultant|F2G: Grant/Research Support|Genovax: Grant/Research Support|Karius: Advisor/Consultant|Merck: Advisor/Consultant|Merck: Grant/Research Support|Pfizer: Grant/Research Support **Erica J. Stohs, MD, MPH**, BioMerieuex: Grant/Research Support|Merck: Grant/Research Support **Catherine Liu, MD**, Pfizer: Grant/Research Support

